# Chronically dysregulated corticosterone impairs dopaminergic transmission in the dorsomedial striatum by sex-divergent mechanisms

**DOI:** 10.1038/s41386-023-01551-1

**Published:** 2023-02-21

**Authors:** Ashley L. Holloway, Michael D. Schaid, Talia N. Lerner

**Affiliations:** 1 Department of Neuroscience, Northwestern University Feinberg School of Medicine, Chicago, IL 60611 USA; 2 Northwestern University Interdepartmental Neuroscience Program (NUIN), Evanston, IL 60208 USA

**Keywords:** Motivation, Cellular neuroscience, Behavioural methods

## Abstract

Major depressive disorder (MDD) is a leading cause of disability worldwide. Individuals with MDD exhibit decreased motivation and deficits in reward processing. In a subset of MDD patients, chronic dysregulation of the hypothalamic-pituitary-adrenal (HPA) axis occurs, resulting in increased levels of the ‘stress hormone’ cortisol during the normal rest period (i.e., evening and night). However, the mechanistic relationship between chronically elevated resting cortisol and behavioral deficits in motivation and reward processing remains unclear. Given that women are diagnosed with MDD at twice the rate of men, it is important to understand whether the mechanisms linking cortisol to the symptoms of MDD differ by sex. In this study, we used subcutaneous implants to chronically elevate free plasma corticosterone (the rodent homolog of cortisol; ‘CORT’) during the rest period in male and female mice and examined changes in behavior and dopamine system function. We found that chronic CORT treatment impaired motivated reward-seeking in both sexes. In female but not male mice, CORT treatment reduced dopamine content in the dorsomedial striatum (DMS). In male but not female mice, CORT treatment impaired the function of the dopamine transporter (DAT) in DMS. From these studies, we conclude that chronic CORT dysregulation impairs motivation by impairing dopaminergic transmission in the DMS, but via different mechanisms in male and female mice. A better understanding of these sex-specific mechanisms could lead to new directions in MDD diagnosis and treatment.

## Introduction

Major depressive disorder (MDD) is a leading cause of disability worldwide, affecting an estimated 5% of adults [1]. Individuals with MDD exhibit decreased motivation and deficits in reward processing [2, 3]. One important factor that precipitates and exacerbates MDD is stress [4]. CORT (corticosterone in rodents, cortisol in humans) is the body’s primary stress hormone, released by the adrenal gland both in a regular circadian rhythm and in response to stressful events. In a subset of individuals with MDD, the circadian regulation of CORT is altered, with chronically elevated levels observed during the rest period (i.e., evening and night) [5, 6]. Increased resting period CORT is particularly evident in psychotic and melancholic depression, and is associated with symptoms of anhedonia and general distress [7–13]. However, it remains unclear how this CORT dysregulation contributes to MDD symptomology.

In rodent preclinical models, chronic elevation of circulating CORT impairs operant responding for rewards, suggesting that elevated CORT may cause impaired reward processing in humans [14, 15]. However, rodent studies have only been carried out in males, leaving open the question of sex differences in the effects of dysregulated CORT. Since MDD is twice as common in women vs men, sex differences in biological responses to dysregulated CORT are important to assess. Furthermore, the biological mechanisms underlying CORT-induced impairments in operant responding in either sex remain unclear.

We hypothesized that CORT dysregulation impacts operant responding by altering dopaminergic transmission. Dopaminergic transmission in the striatum regulates reward processing, motivation, and associative learning [16–20]. Dopaminergic transmission within the striatum occurs in two modes: tonic and phasic [21, 22]. Tonic dopamine is the sustained level of extracellular dopamine in the striatum. It arises from the tonic firing activity of dopamine neurons and is also tightly regulated by dopamine reuptake into terminals by the dopamine transporter, DAT [23, 24]. Tonic dopamine is hypothesized to govern motivation [25, 26]. Phasic dopamine transmission occurs when dopamine neurons fire bursts of action potentials in discrete epochs on top of tonic dopamine. Phasic dopamine transients facilitate associative learning about cues and actions that precede rewards [27–30]. We examined whether impaired operant responding for rewards following chronic CORT treatment was associated with impaired tonic and phasic dopaminergic transmission in two striatal subregions critical for effortful operant responding: the nucleus accumbens core (NAcc) and the dorsomedial striatum (DMS).

## Methods

### Animals & housing

Adult (10 + weeks) male and female C57BL6/J mice were group-housed by sex and treatment (2–5 mice per cage) and given ad libitum access to food and water, unless otherwise specified. Mice were housed on a 14:10 h light/dark cycle, in a temperature- and humidity-controlled environment. All experimental procedures were approved by the Northwestern University Animal Care and Use Committee. All experiments were completed at zeitgeber time 4–6 (4–6 h after lights-on).

### Subcutaneous pellet implants

At 10+ weeks of age, mice were anesthetized with isoflurane and given analgesics to minimize pain after surgery. Hair was removed from the lateral portion of the neck using Nair, and the skin was swabbed with alcohol and iodine. A small incision was made, and Placebo or Corticosterone (35 mg; 60-day release; Innovative Research of America) slow-release pellets were implanted subcutaneously in the space between the shoulder and neck. The incision was closed with non-absorbable sutures. For ex vivo slice imaging and in vivo photometry experiments, pellets were implanted during stereotaxic surgeries.

### Stereotaxic surgeries

At 10+ weeks of age, mice were anesthetized with isoflurane and given analgesics to minimize pain. Hair on the skin of the top of the head was removed using Nair, then swabbed with alcohol and iodine. A single incision was made down the midline of the skull, then a hole was drilled above the injection site for the dorsomedial striatum (DMS; + 0.8 A/P, 1.5 M/L, -2.8 D/V, relative to bregma) and nucleus accumbens core (NAcc: +1.6 A/P, 0.8 M/L, -4.1 D/V). 500 nL of AAV9-CAG-dLight1.3b (7 × 10^11^ VG/mL) [31] was injected into the DMS and NAcc at a rate of 100 nL/min using a Hamilton syringe. The needle remained in place for five minutes after injection before being slowly retracted. For fiber photometry experiments, a fiber optic (Doric, 400 µm core, 0.66 NA) was implanted over the DMS injection site. The hemispheres of injection sites were counterbalanced across treatment groups and sexes.

### Operant conditioning

Mice were food restricted to 85% of their ad libitum weight and monitored for maintenance of this weight throughout operant training. Operant sessions lasted 60 min, or until mice received the maximum number of rewards available (50 rewards). Mice were initially trained to acquire sucrose rewards from the reward port of an operant box (Med Associates) in the absence of any contingency. Mice then advanced to a fixed-ratio (FR) schedule of training during which they had to nosepoke once for one sucrose pellet (FR-1). After earning at least 30 rewards for two consecutive days (criterion for advancement), mice were advanced to FR-3 training, in which they had to nosepoke three times for one sucrose pellet. After reaching criterion for advancement, mice were advanced to FR-5 training.

#### High performance liquid chromatography and electrochemical detection of dopamine

Biogenic amines were measured in the Vanderbilt University Neurochemistry Core.

### Ex vivo dLight1.3b imaging

At least 4 weeks after pellet implantation and stereotaxic surgery, mice were anesthetized with Euthasol (Virbac, 1 mg/kg) and transcardially perfused with ice-cold N-methyl-D-glucamine (NMDG) [32] artificial cerebrospinal fluid (ACSF). Coronal tissue sections (300 µm thick) containing the DMS were cut using a vibratome (Leica VT1200) and transferred to NMDG ACSF at 33 °C. Slices recovered in HEPES ACSF and holding ACSF, as described previously [32, 33]. All solutions were saturated with carbogen (95% Oxygen, 5% Carbon Dioxide) and their pH and osmolarity were adjusted to 7.3–7.4 and 300 ± 5 mOsm, respectively. Slices were transferred to a recording chamber in ACSF, held at 30–32 °C. For recording, ACSF contained blockers for AMPARs (NBQX, 5 µM), NMDARs (D-AP5, 50 µM), nAChRs (DHβE, 1 µM), GABA_A_Rs (Picrotoxin, 50 µM), and GABA_B_Rs (CGP-54626, 2 µM). Dopamine release was evoked using a bipolar stimulating electrode (FHC, Inc.) placed ~300 microns from the imaging site. All stimulations were 4 V, with a pulse width of 0.5 ms. After baseline recordings, the DAT inhibitor GBR-12909 (1 µM) was applied to slices, followed by the OCT3 inhibitor Normetanephrine (50 µM). dLight1.3b fluorescence was imaged using a scientific CMOS camera (Hamamatsu Orca-Flash 4.0LT), with a sampling rate of 33 Hz. dLight1.3b tau-off values were calculated using a custom MATLAB script.

### In vivo dLight1.3b fiber photometry

Fiber photometry experiments occurred at least four weeks after pellet implantation and stereotaxic surgeries. Mice were attached to a fiber optic patch cord (Doric, 400 µm core, 0.66 NA) and gently placed in an open field (28 × 28 cm). After 10 min in the open field, mice were injected with the DAT inhibitor, GBR-12909 (20 mg/kg), and returned to the open field for another 40 min. Fiber photometry data was collected throughout the entire time that mice were in the open field. Data acquisition and processing details are described in the supplement. GuPPy, an open-source Python-based photometry data analysis pipeline, was used to determine dLight1.3b transient timepoints [34]. A custom MATLAB script was used to calculate dLight1.3b area-under-the-curve (AUC). Locomotor activity was recorded using Noldus Ethovision XT 16.

### Western blot

An equal amount of protein from each sample was loaded in a Tris-Glycine gel (Invitrogen). Protein was transferred to a PVDF membrane and blocked in either 5% bovine serum albumin (BSA) in Tris-buffered saline + 0.1% Tween-20 (TBS-T) for phospho-DAT, or 5% non-fat milk (NFM) in TBS-T for DAT and Beta-Actin. Membranes were blocked for one hour at room temperature, then incubated in primary antibody in blocking buffer overnight at 4 °C. Membranes were washed in TBS-T, then incubated in secondary antibody in blocking buffer for 1–2 h at room temperature. Membranes were imaged using a Licor Odyssey Fc Imaging System. Densitometric analysis was completed using ImageJ. Protein expression was normalized to the average of the sex-matched Placebo group for statistical analysis.

Additional methods are provided in the supplement.

## Results

### Chronic corticosterone (CORT) treatment increases total plasma CORT in male mice and decreases plasma corticosteroid binding globulin (CBG) levels in both sexes

**Fig. 1:**
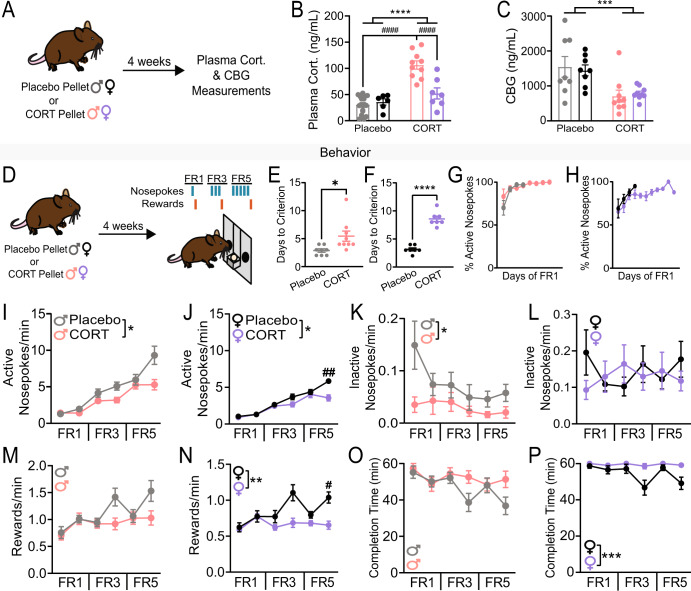
Chronic corticosterone treatment increases plasma CORT levels in males, decreases plasma CBG levels in both sexes, and impairs motivation. **A** Experimental timeline for pellet implantation and plasma CORT and CBG measurements. **B** Plasma corticosterone (ng/mL) in male and female mice implanted with a placebo or corticosterone (35 mg; CORT) pellet. Two-way ANOVA, main effect of treatment ****p < 0.0001, main effect of sex *p* < 0.01, main effect of treatment x sex interaction *p* < 0.001, multiple comparisons ^####^*p* < 0.0001. **C** Plasma CBG (ng/mL) in male and female mice implanted with a placebo or corticosterone (35 mg; CORT) pellet. Two-way ANOVA, main effect of treatment ***p < 0.001. **D** Experimental timeline for pellet implantation and operant behavior paradigms with schematic of fixed ratio (FR) paradigms. **E** Days to reach criterion for FR1 in male mice. Unpaired two-tailed t-test *p < 0.05. Each point represents an individual. **F** Days to reach criterion for FR1 in female mice. Unpaired two-tailed t-test ****p < 0.0001. Each point represents an individual. **G** Percent active nosepokes over days of FR1 until criterion was met in Placebo- (grey) and CORT-treated (pink) male mice. Each point represents mean ± SEM percent active nosepokes for a given day of FR1 training. **H** Percent active nosepokes over days of FR1 until criterion was met in Placebo- (black) and CORT-treated (purple) female mice. Each point represents mean ± SEM percent active nosepokes for a given day of FR1 training. **I** Active nosepoking rates of Placebo- (*N* = 8) and CORT- (*N* = 9) treated male mice across operant behavior paradigms. Two-way ANOVA, main effect of treatment *p < 0.05, main effect of treatment x paradigm interaction *p* < 0.001. **J** Active nosepoking rates of Placebo- (*N* = 7) and CORT- (*N* = 7) treated female mice across operant behavior paradigms. Two-way ANOVA, main effect of treatment *p < 0.05, main effect of treatment x paradigm interaction *p* < 0.001. **K** Inactive nosepoking rates of Placebo- and CORT-treated male mice across operant behavior paradigms. Two-way ANOVA, main effect of treatment *p < 0.05. **L** Inactive nosepoking rates of Placebo- and CORT-treated female mice across operant behavior paradigms. **M** Reward rates of Placebo- and CORT-treated male mice across operant behavior paradigms. **N** Reward rates of Placebo- and CORT-treated female mice across operant behavior paradigms Two-way ANOVA, main effect of treatment **p < 0.01, main effect of treatment x paradigm interaction *p* < 0.01, multiple comparisons ^#^*p* < 0.05. **O** Time to completion of operant session (in minutes) for Placebo- and CORT-treated male mice across operant behavior paradigms. Two-way ANOVA, main effect of treatment x paradigm interaction *p* < 0.05. **P** Time to completion of operant sessions (in minutes) for Placebo- and CORT-treated female mice across operant behavior paradigms. Two-way ANOVA, main effect of treatment ***p < 0.001, main effect of treatment x paradigm interaction *p* < 0.05. Data presented as mean ± SEM.

To chronically elevate plasma CORT levels during the rest period, we implanted male and female mice with subcutaneous slow-release CORT pellets (35 mg, 60-day release); control groups received Placebo pellets of the same size. Slow-release pellets were used to increase circulating CORT levels during the rest period (the light phase for mice), thereby disrupting circadian rhythms of CORT [35, 36] as observed in some individuals with MDD [5, 6]. This approach differs from another commonly used approach, CORT administration via drinking water, which preferentially increases circulating CORT levels during the active phase, when mice drink more often [37]. To test if slow-release CORT pellet treatment chronically elevated plasma CORT levels during the rest period, we collected blood from Placebo- and CORT-treated mice at zeitgeber time 4-6 (ZT4-6, 4–6 h after lights on) four weeks after implantation and used an enzyme-linked immunosorbent assay (ELISA) to quantify total plasma CORT (Fig. 1A). There was a significant effect of treatment (Two-way ANOVA, *F*_(1, 37)_=41.18, *p* < 0.0001), a significant effect of sex (*F*_(1,37)_ = 11.78, *p* < 0.01), and a significant interaction between treatment and sex (*F*_(1,37)_ = 17.25, *p* < 0.001). Notably, we found that CORT pellet implant increased total plasma CORT in male mice only and resulted in higher levels of resting CORT in male vs female mice (Placebo Male vs CORT Male, Tukey’s multiple comparisons *p* < 0.0001; CORT Male vs CORT Female, *p* < 0.0001; Fig. 1B). This sex difference in total plasma CORT four weeks after pellet implantation is consistent with previous studies in rats [38], and likely occurs due to sex differences in hypothalamic-pituitary-adrenal (HPA) axis responsivity [39]. However, a limitation of measuring total plasma CORT is that it includes both free and protein-bound CORT. Free CORT can cross the blood-brain barrier, while protein-bound CORT cannot [40–42]. Corticosteroid binding globulin (CBG) is the primary blood protein that binds CORT. Thus, we questioned if chronic CORT treatment decreased CBG, which could augment circulating levels of free CORT, even in the absence of changes in total levels. Chronic CORT treatment decreased CBG levels with no evidence of sex difference (Fig. 1C; Two-way ANOVA, significant effect of treatment, *F*_(1,30)_=16.30, *p* < 0.001; no sex x treatment interaction). Treatment did not affect estrous cyclicity of females (Fig. S1). We concluded that circulating levels of free CORT are likely elevated in both male and female mice after treatment with subcutaneous slow-release CORT pellets but to differing degrees of severity. Due to the significant sex difference in plasma CORT levels after CORT treatment, we separated the sexes for analysis in all following experiments.

### Chronic CORT treatment impairs motivated reward-seeking in male and female mice

Chronic CORT treatment has previously been shown to impair reward-seeking behaviors in male mice [14, 15]. However, it was unclear what effect chronic CORT treatment would have on female mice. To assess reward-seeking behaviors in both sexes, we used operant training. Four weeks after Placebo or CORT pellet implantation, mice began training on a fixed ratio-1 (FR-1) schedule, then advanced to FR-3 and FR-5 (Fig. 1D). We found that CORT treatment significantly increased the number of days it took both sexes to reach criterion on the FR-1 task (Fig. 1E, F; Unpaired two-tailed t-test, *p* < 0.05 male, *p* < 0.0001 female). However, CORT-treated mice readily learned the association between the active nosepoke and reward. CORT- and Placebo-treated mice similarly discriminated between the active and inactive nosepokes during the initial days of FR-1 training (Fig. 1G, H), but CORT-treated mice were slower to use this associative knowledge to reach the criterion of obtaining 30 rewards per session. This finding suggests that CORT-treated mice have intact reward learning but are less motivated to attain rewards than Placebo-treated mice. After FR-1 criterion was met, CORT-treated mice exhibited decreased rates of nosepoking across FR-3 and FR-5 sessions (Fig. 1I, J; males: significant effects of treatment [Two-way ANOVA *F*_(1,15)_ = 5.554, *p* < 0.05], day of training [*F*_(2.513,37.70)_ = 37.70, *p* < 0.0001], and an interaction between treatment and day of training [*F*_(5,75)_ = 4.749, *p* < 0.001]; females: significant effects of treatment [Two-way ANOVA *F*_(1,12)_ = 5.098, *p* < 0.05], day of training [*F*_(3.267,39.20)_ = 87.47, *p* < 0.0001], and an interaction between treatment and day of training [Two-way ANOVA *F*_(5,60)_ = 7.294, *p* < 0.0001]). Again, no deficit in active nosepoke discrimination was observed (in fact, CORT-treated males made significantly fewer inactive nosepokes than Placebo-treated males, Fig. 1K, L; males: Two-way ANOVA, significant effect of treatment [*F*_(1,15)_=6.123, *p* < 0.05]; females: no significant effect of treatment). Therefore, as for initial FR1 training, decreased rates of active nosepoking in CORT-treated mice do not stem from impaired learning, but likely arise due to decreased motivation. Motivational deficits in CORT-treated mice are further supported by their impaired rate of rewards earned, particularly on the final days of FR-3 and FR-5 (Fig. 1M, N; males: Two-way ANOVA, significant effects of day of training [*F*_(3.422,51.34)_ = 7.968, *p* < 0.0001], and an interaction between day of training and treatment [*F*_(5,75)_=3.320, *p* < 0.01); females: Two-way ANOVA, significant effects of treatment [*F*_(1,12)_=16.15, *p* < 0.01], day of training [*F*_(3.275,39.30)_=5.835, *p* < 0.01], and an interaction between day of training and treatment [*F*_(5,60)_ = 4.427, *p* < 0.01]). While CORT-treated males earned the same number of rewards as Placebo-treated males across operant training (Fig. S2A), they took longer to earn those rewards, especially on the final days of FR-3 and FR-5 (Fig. 1O; Two-way ANOVA, significant effect of an interaction between treatment and day of training, *p* < 0.01). CORT-treated females also took longer to earn rewards (Fig. 1P; Two-way ANOVA, significant effects of treatment [*F*_(1,12)_ = 19.20, *p* < 0.001], day of training [*F*_(3.360,40.32)_ = 3.978, *p* < 0.05], and an interaction between treatment and day of training [*F*_(5,60)_ = 2.648, *p* < 0.05]) and earned significantly fewer total rewards (Fig. S2B) than Placebo-treated females. These results suggest continued motivational impairments throughout training. In female mice only, CORT treatment significantly decreased reward port entry rates (i.e., actions to retrieve earned rewards, Fig. S2C, D), suggesting that CORT treatment may also induce anhedonia in females.

### Chronic CORT treatment does not impair phasic dopamine transmission during reward-seeking

After observing an effect of CORT treatment on reward-seeking in both sexes, we questioned if CORT treatment was impairing phasic dopamine transmission in the striatum. To examine phasic dopamine transients in Placebo- and CORT-treated mice, we injected a virus encoding the fluorescent dopamine sensor, dLight1.3b (AAV9-CAG-dLight1.3b), into the NAcc and DMS. We implanted a fiber optic over each injection site and recorded dLight1.3b transients during operant training using fiber photometry. We found that CORT treatment did not affect phasic dLight1.3b transients in the NAcc or DMS (Fig. S3), thus we pursued measures of tonic dopamine activity.

### Chronic CORT treatment decreases tissue dopamine content of the dorsomedial striatum (DMS) in female mice

**Fig. 2:**
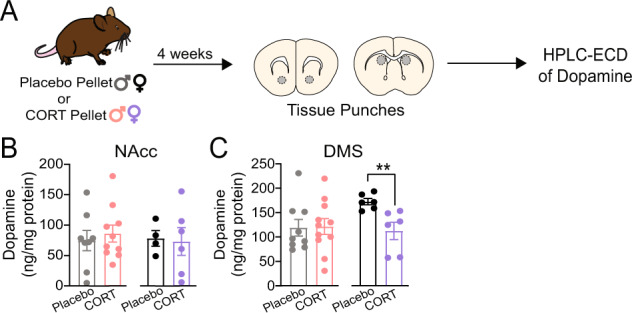
Chronic corticosterone treatment significantly decreases tissue dopamine content of the dorsomedial striatum (DMS) in female mice. **A** Experimental timeline for pellet implantation, tissue punches, and HPLC-ECD of dopamine. **B** Tissue dopamine content of the nucleus accumbens core (NAcc) of placebo- and CORT-treated male and female mice. **C** Tissue dopamine content of the DMS of placebo- and CORT-treated male and female mice. Unpaired two-tailed t-test, **p < 0.01. Each point represents an individual. Data presented as mean ± SEM.

To investigate whether chronic CORT treatment influenced dopamine content of the striatum, we analyzed tissue samples from the NAcc and DMS of Placebo- and CORT-treated mice using high-performance liquid chromatography and electrochemical detection (HPLC-ECD) of dopamine. CORT treatment did not affect NAcc dopamine content in either sex (Fig. 2B), but significantly decreased DMS dopamine content in female mice (Fig. 2C; Unpaired two-tailed t-test, *p* < 0.01). Therefore, although acute CORT has effects on NAcc dopamine [43], DMS dopamine is more sensitive to chronic CORT treatment in females.

### Chronic CORT treatment impairs dopamine transporter (DAT) function in the DMS of male mice

**Fig. 3:**
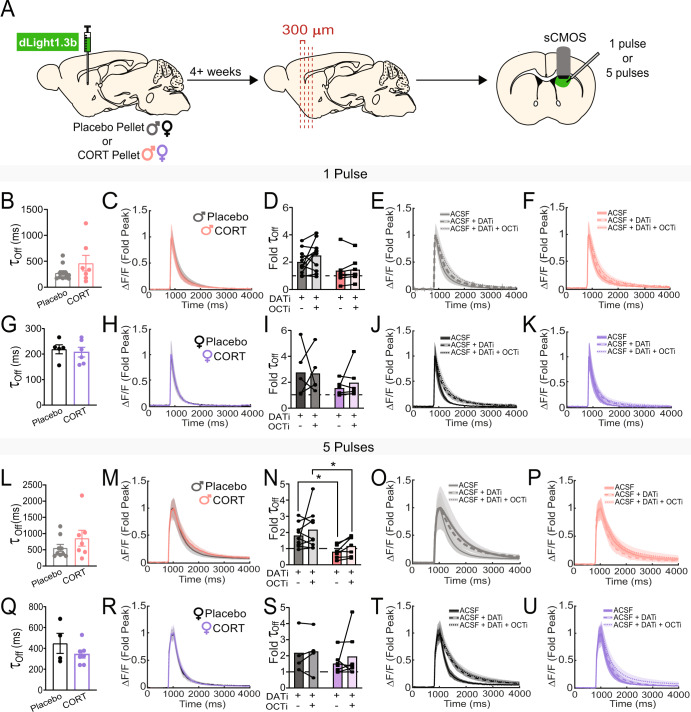
Chronic corticosterone treatment impairs ex vivo dopamine transporter (DAT) function in the dorsomedial striatum (DMS) of male mice. **A** Experimental timeline for viral injection, pellet implantation, and slice imaging experiments. **B** dLight1.3b fluorescence tau-off values after a single electrical stimulation of the DMS in acute tissue slices from male mice. **C** Average dLight1.3b fluorescence traces, normalized to the peak of dLight1.3b fluorescence. after a single electrical stimulation of the DMS in acute tissue slices from male mice. **D** Fold change of tau-off values of dLight1.3b fluorescence in the presence of inhibitors for the dopamine transporter (DATi) and organic cation transporter 3 (OCTi), normalized to tau-off values of dLight1.3b fluorescence in the absence of any transporter inhibitors, after a single electrical stimulation of the DMS in acute tissue slices from male mice. Two-Way ANOVA, trending effect of treatment *p* = 0.07. **E**, **F** Average dLight1.3b fluorescence traces, normalized to the peak of dLight1.3b fluorescence, after a single electrical stimulation of the DMS in acute slices from Placebo- (**E**) and CORT- (**F**) treated male mice, in the presence and absence of DATi and OCTi. **G** dLight1.3b fluorescence tau-off values after a single electrical stimulation of the DMS in acute tissue slices from female mice. **H** Average dLight1.3b fluorescence traces, normalized to the peak of dLight1.3b fluorescence, after a single electrical stimulation of the DMS in acute tissue slices from female mice. **I** Fold change of tau-off values of dLight1.3b fluorescence in the presence of DATi and OCTi, normalized to tau-off values of dLight1.3b fluorescence in the absence of any transporter inhibitors, after a single electrical stimulation of the DMS in acute tissue slices from female mice. **J**, **K** Average dLight1.3b fluorescence traces, normalized to the peak of dLight1.3b fluorescence, after a single electrical stimulation of the DMS in acute tissue slices from Placebo- (**J**) and CORT- (**K**) treated female mice, in the presence and absence of DATi and OCTi. **L** dLight1.3b fluorescence tau-off values after a 20 Hz, 5 pulse electrical stimulation of the DMS in acute tissue slices from male mice. **M** Average dLight1.3b fluorescence traces, normalized to the peak of dLight1.3b fluorescence, after a 20 Hz, 5 pulse electrical stimulation of the DMS in acute tissue slices from male mice. **N** Fold change of tau-off values of dLight1.3b fluorescence in the presence of DATi and OCTi, normalized to tau-off values of dLight1.3b fluorescence in the absence of any transporter inhibitors, after a 20 Hz, 5 pulse electrical stimulation of the DMS in acute tissue slices from male mice. Two-Way ANOVA, main effect of treatment *p* < 0.05, multiple comparisons *p < 0.05. **O**, **P** Average dLight1.3b fluorescence traces, normalized to the peak of dLight1.3b fluorescence, after a 20 Hz, 5 pulse electrical stimulation of the DMS in acute tissue slices from Placebo- (**O**) and CORT- (**P**) treated male mice, in the presence and absence of DATi and OCTi. **Q** dLight1.3b fluorescence tau-off values after a 20 Hz, 5 pulse electrical stimulation of the DMS in acute tissue slices from female mice. **R** Average dLight1.3b fluorescence traces, normalized to the peak of dLight1.3b fluorescence, after a 20 Hz, 5 pulse electrical stimulation of the DMS in acute tissue slices from female mice. **S** Fold change of tau-off values of dLight1.3b fluorescence in the presence of DATi and OCTi, normalized to tau-off values of dLight1.3b fluorescence in the absence of any transporter inhibitors, after a 20 Hz, 5 pulse electrical stimulation of the DMS in acute tissue slices from female mice. **T**, **U** Average dLight1.3b fluorescence traces, normalized to the peak of dLight1.3b fluorescence, after a 20 Hz, 5 pulse electrical stimulation of the DMS in acute tissue slices from Placebo- (**T**) and CORT- (**U**) treated female mice, in the presence and absence of DATi and OCTi. Points represent the average of 2–3 sweeps from a single individual. Data presented as mean ± SEM.

One mechanism that regulates levels of tonic dopamine in the striatum is modulation of dopamine transporter (DAT) function [24]. By altering decay rates of phasic dopamine transients, changes in DAT function can alter the timescale for integration of phasic dopamine signals, allowing or disallowing the buildup of tonic levels when dopamine neurons are active. Chronic DAT impairment can also cause compensation in the dopamine system, altering the rate of synthesis of new dopamine [24]. To investigate DAT function in mice chronically treated with CORT, we assayed dopamine dynamics in an ex vivo slice preparation. We injected a virus encoding the fluorescent dopamine sensor, dLight1.3b (AAV9-CAG-dLight1.3b), into the NAcc and DMS, and implanted Placebo or CORT pellets during the same surgery. Four weeks later, we prepared striatal tissue sections and electrically evoked dopamine release while imaging dLight1.3b fluorescence (Fig. 3A, Fig. S4). To mimic tonic and phasic dopamine neuron firing, we used a single stimulation pulse or a burst of 5 pulses at 20 Hz, respectively. We quantified the decay of evoked dLight1.3b transients by calculating a ‘tau-off’ value and used it as a measure of the speed of extracellular dopamine clearance [31, 44]. CORT treatment did not significantly increase tau-off in male (Fig. 3B, L) or female (Fig. 3G, Q) mice in response to either one or five pulses at baseline in the DMS or NAcc (Fig. S5). However, the lack of change could be due to compensation for chronic DAT impairment. To elucidate how DAT activity was contributing to tau-off, we washed a DAT inhibitor, GBR12909 (1 µM, ‘DATi’), onto the slice. We also tested the contribution of another monoamine transporter, Organic Cation Transporter 3 (OCT3) [45], to dopamine clearance by washing on an OCT3 inhibitor, normetanephrine (50 µM; ‘OCTi’). OCT3 is a low-affinity, high-capacity non-specific monoamine transporter [46]. Although OCT3 does not regulate synaptic dopamine levels as effectively as DAT, CORT binds OCT3 directly and inhibits reuptake, making it important to examine in our studies [45, 47–49]. In the NAcc, we did not observe any significant effect of CORT treatment on tau-off after DAT and OCT3 inhibition in either sex (Fig. S5). Using one stimulation pulse in the DMS, we did not observe significant differences in tau-off between Placebo- and CORT-treated mice after DAT and OCT3 inhibition (Fig. 3D–F, I–K), although there was a trending effect of CORT treatment in male mice (Fig. 3D, Two-way ANOVA, *F*_(1,16)_=3.636, p = 0.07). In response to five pulses in the DMS, DAT inhibition slowed dopamine clearance in Placebo-treated males, but had no effect in CORT-treated males, indicating DAT function is impaired in the DMS of CORT-treated males (Fig. 3N–P; Two-way ANOVA, significant effect of treatment, *F*_(1,14)_ = 8.566, *p* < 0.05). In females, CORT treatment did not impair DAT or OCT3 function in the DMS (Fig. 3S–U). CORT treatment did not affect the amplitude of dLight1.3b fluorescence elicited by one or five stimulation pulses in the DMS of either sex (Fig. S6).

**Fig. 4:**
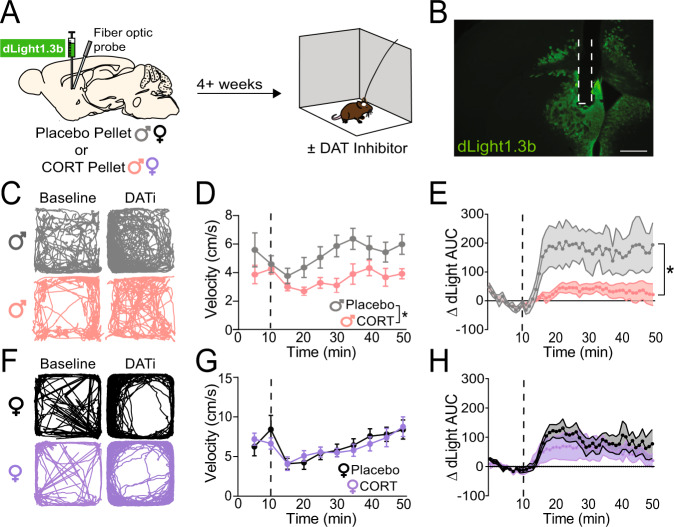
Chronic corticosterone treatment impairs in vivo dopamine transporter (DAT) function in the dorsomedial striatum (DMS) of male mice. **A** Experimental timeline for viral injection, fiber optic implant, pellet implantation, and open field behavior. **B** Representative image of dLight1.3b viral spread and fiber optic implantation site (outlined in dashed white line). Scale bar equals 500 micrometers. **C** Representative activity traces of male mice (Placebo, top; CORT, bottom) during the ten-minute baseline period (‘Baseline’) and the last ten minutes of recorded activity after injection of the DAT inhibitor GBR12909 (20 mg/kg, i.p., ‘DATi’). **D** Velocity of Placebo- (*N* = 10) and CORT- (*N* = 9) treated male mice, in averaged five-minute bins, before and after injection with the DAT inhibitor, GBR12909 (20 mg/kg, i.p.; injection time indicated by vertical dashed line). Two-Way ANOVA, main effect of treatment *p < 0.05, trending effect of time *p* = 0.06. **E** Change in dLight1.3b area-under-the-curve (AUC) relative to the minute average of the ten-minute baseline period prior to injection with the DAT inhibitor, GBR12909 (20 mg/kg, i.p.; injection time indicated by vertical dashed line) in male mice. Two-Way ANOVA, main effect of treatment *p < 0.05, main effect of time *p* < 0.01, main effect of treatment x time interaction *p* < 0.0001. Placebo *N* = 5, CORT *N* = 6. **F** Representative activity traces of female mice (Placebo, top; CORT, bottom) during the ten-minute baseline period (‘Baseline’) and the last ten minutes of recorded activity after injection of the DAT inhibitor GBR12909 (20 mg/kg, i.p., ‘DATi’). **G** Velocity of Placebo- (*N* = 6) and CORT- (*N* = 8) treated female mice, in averaged five-minute bins, before and after injection with the DAT inhibitor, GBR12909 (20 mg/kg, i.p.; injection time indicated by vertical dashed line). Two-Way ANOVA, main effect of time *p* < 0.001. **H** Change in dLight1.3b AUC relative to the average of the ten-minute baseline period prior to injection with the DAT inhibitor, GBR12909 (20 mg/kg, i.p.; injection time indicated by vertical dashed line) in female mice. Two-Way ANOVA, main effect of time *p* < 0.01. Placebo *N* = 5, CORT *N* = 6. Data presented as mean ± SEM.

To verify our ex vivo results, we designed an experiment to examine DAT function in vivo using fiber photometry. We injected a virus encoding dLight1.3b (AAV9-CAG-dLight1.3b) into the DMS of male and female mice and implanted a fiber optic in DMS for in vivo recording during behavior (Fig. S7). Four weeks later, mice were placed in an open field to collect baseline locomotor and dLight1.3b fluorescence data (Fig. 4A). After ten minutes of baseline data collection, mice were injected with the DAT inhibitor, GBR12909 (20 mg/kg, i.p.), and returned to the open field for forty minutes. In mice with high DAT activity, injection of a DAT inhibitor should increase locomotion, with a concordant increase in extracellular dopamine in DMS (measured as a change in the dLight1.3b fluorescence area-under-the-curve (AUC)). After DAT inhibition, locomotion of CORT-treated male mice was blunted relative to Placebo-treated male mice (Fig. 4D; Two-way ANOVA, significant effect of treatment [*F*_(1,17)_ = 6.776, *p* < 0.05], trending effect of time [*F*_(2.344,39.86)_ = 2.835, *p* = 0.06]). Furthermore, CORT-treated male mice exhibited significant blunting of dLight1.3b AUC after DAT inhibition compared to Placebo-treated male mice (Fig. 4E; Two-way ANOVA, significant effects of treatment [*F*_(1,9)_ = 5.418, *p* < 0.05], time [*F*_(2.439,21.95)_ = 7.083, *p* < 0.01], and the interaction between treatment and time [*F*_(49,441)_ = 2.979, *p* < 0.0001]).

DAT inhibition could increase dLight1.3b AUC by increasing the decay constant of dLight1.3b transients, leading to a larger AUC per transient. Longer dopamine clearance times (indicated by higher decay constants) could then slowly increase baseline dLight1.3b fluorescence, reflecting a slow buildup of tonic dopamine. Such an increase in baseline fluorescence would also contribute to an increase in dLight1.3b AUC following DAT inhibition. To differentiate between these two effects, we analyzed the decay constants of dLight1.3b transients recorded during open field behavior before and after DAT inhibition. We found that DAT inhibition increased both the decay time constants of in vivo dLight1.3b transients and led to a buildup of baseline dLight1.3b fluorescence (Fig. S8). We observed non-significant trends in which both of these effects were greater in Placebo-treated males than CORT-treated males (Fig. S8; Two-way ANOVA, effect of treatment *p* = 0.06 for decay constants, *p* = 0.08 for baseline fluorescence). Thus, we concluded that the significant difference in DMS dLight1.3b AUC between Placebo- and CORT-treated males after DAT inhibition (Fig. 4E) is the result of a *combined* effect on the decay of individual dLight1.3b transients and an increase in the baseline dLight1.3b fluorescence due to integration of slowly decaying transients.

In female mice, we did not observe differences between treatment conditions (Fig. 4F–H). We observed a significant effect of time on locomotion (Fig. 4G; Two-way ANOVA, *F*_(2.062,24.75)_ = 9.222, *p* < 0.001) and on dLight1.3b AUC (Fig. 4H; Two-way ANOVA, *F*_(2.642,23.78)_ = 7.124, *p* < 0.01) after DAT inhibition in both Placebo- and CORT-treated groups. We concluded that CORT treatment impairs DAT function in the DMS of male, but not female, mice. However, from these data it was unclear *how* CORT treatment impaired DAT function.

### Chronic CORT treatment decreases phosphorylation of DAT at threonine-53

**Fig. 5:**
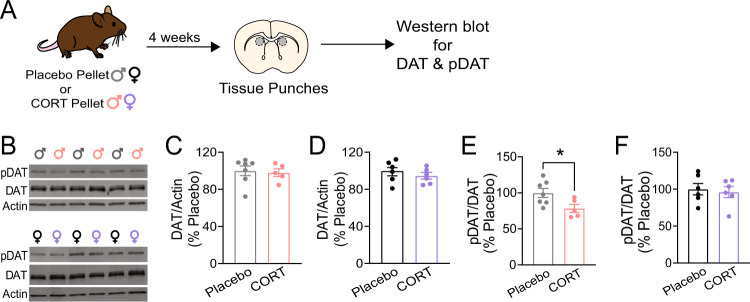
Chronic corticosterone treatment decreases phosphorylation of the dopamine transporter (DAT) at threonine-53 in the dorsomedial striatum (DMS) of male mice. **A** Experimental timeline for pellet implantation, tissue punches, and western blot experiments. **B** Representative western blots for phosphorylated DAT at threonine-53 (‘pDAT’), DAT, and beta-actin (‘Actin’) from DMS tissue samples of male mice (top) and female mice (bottom). **C** Membrane-bound DAT expression, normalized to Actin and plotted as a percent of Placebo expression, from DMS tissue samples of male mice. **D** Membrane-bound DAT expression, normalized to Actin and plotted as a percent of Placebo expression, from DMS tissue samples of female mice. **E** pDAT expression, normalized to DAT and plotted as a percent of Placebo expression, from DMS tissue samples of male mice. Unpaired two-tailed t-test *p < 0.05. **F** pDAT expression, normalized to DAT and plotted as a percent of Placebo expression, from DMS tissue samples of female mice. Each point represents a single individual. Data presented as mean ± SEM.

To assess how CORT treatment impaired DAT function, we examined DAT expression and post-translational modifications of DAT, which regulate reuptake activity [24, 50, 51]. Specifically, we examined phosphorylation at threonine-53, a known regulatory site [51–54]. We collected DMS tissue punches from Placebo- and CORT-treated male and female mice and fractionated the tissue homogenate to isolate membrane-bound proteins. We then performed western blots probing for DAT and Thr53 phospho-DAT (pDAT). We found that CORT treatment had no effect on total levels of membrane-bound DAT in males or females (Fig. 5C, D). However, CORT treatment significantly decreased pDAT in male mice (Unpaired two-tailed t-test, *p* < 0.05), but not female mice (Fig. 5E, F). These results suggest that CORT treatment impairs DMS DAT function in male mice by decreasing phosphorylation of DAT at threonine-53, and further supports the conclusion that DMS DAT is unaffected by CORT treatment in female mice.

## Discussion

Previous studies in rodents have shown that chronic dysregulation of circulating CORT – a condition that also occurs in subsets of human MDD patients – impairs reward processing, but there was little mechanistic insight into *how* CORT dysregulation impairs reward processing. Further, preclinical literature previously reported effects of CORT on operant responding for rewards only in male rodents, yet humans with MDD are majority female. Here, we specifically set out to study both male and female mice and to identify mechanisms by which chronic CORT dysregulation might impact dopaminergic transmission, which is known to underlie operant responding for rewards. We found that chronic CORT treatment impairs motivation to attain rewards in operant paradigms in both male and female mice (Fig. 1I, J), but by sex-divergent mechanisms. In females, CORT treatment decreases tissue dopamine content in the dorsomedial striatum (DMS; Fig. 2C). In males, CORT treatment impairs dopamine transporter (DAT) function in DMS (Figs. 3–5). Despite differing mechanisms, both males and females experienced changes in dopaminergic transmission specifically in DMS, tying dopaminergic function in this striatal subregion to the observed deficits in motivation. This discovery is consistent with studies showing that DMS dopamine governs goal-directed operant responding for rewards and tying tonic dopamine to motivation [18, 19, 25, 26, 55]. Critically, chronic CORT treatment did not affect dopaminergic transmission in the NAcc, consistent with reports that adrenalectomy and CORT replacement do not affect NAcc extracellular dopamine levels [56].

Our discovery of a latent sex difference in the mechanism by which DMS dopamine transmission is affected by CORT treatment adds to a growing body of literature indicating that males and females can display different underlying mechanisms to achieve similar functional or behavioral outcomes [57, 58]. Therefore, it is important not to assume that a lack of observed sex differences at a high level of analysis precludes sex differences in mechanism. Indeed, we must continue to probe for sex differences at the molecular level if we are to appropriately translate preclinical discoveries into medicines that act at the molecular level.

Based on our results, we speculate that reward processing deficits observed in MDD patients with dysregulated CORT may similarly be due to impaired DMS dopamine transmission, caused by distinct mechanisms in males and females. Our speculation is consistent with recent studies showing that individuals with MDD exhibit decreased DAT expression and tonic dopamine within the dorsal striatum [3, 59]. However, to address the aspect of our hypothesis dealing with sex differences, human data must be analyzed by sex. If the sex-divergent mechanisms by which DMS dopamine transmission is impaired in mice hold true for humans, this would suggest that medications for MDD should be tailored by sex. Further, it may be valuable to distinguish MDD patient populations by phenotyping for CORT dysregulation. Notably, previous failed attempts to translate HPA axis-based therapies from rodent models to humans failed to account for sex differences [39]. They also did not include analyses of CORT status or other aspects of HPA axis function, which could help segregate patient populations most amenable to an HPA axis-focused therapeutic approach.

One caveat of our studies is that the chronic CORT treatment we applied significantly increased total plasma CORT in males only. This finding suggests that CORT elevation drives the behavioral and neurobiological effects observed in males, but it is less clear whether CORT elevation is achieved in females. Free CORT, which crosses the blood-brain barrier, may be increased in females due to lower CBG levels, but this hypothesis is not fully confirmed. The sex difference in total plasma CORT levels in response to CORT pellet implantation is consistent with an extensive literature demonstrating sex differences in feedback regulation of the hypothalamic-pituitary-adrenal (HPA) axis, which may be fundamental to consider in a variety of stress studies, not only ours [39, 60]. Further, at least one clinical report associated lower CBG levels with MDD in female patients only, which our CORT treatment model intriguingly recapitulates [61]. Follow-up studies are necessary to understand how CORT dosage affects total and free plasma CORT levels in males and females. The dose-response effects of CORT on behavior are known to follow an ‘inverted U’ wherein levels of CORT that are either too high or too low are problematic [62]. While we focused these studies on understanding the effects of elevated CORT, on the downswing of the inverted U, clinical studies have also found that some individuals with MDD exhibit insufficient CORT levels [63]. Therefore, understanding the dose-response relationship between CORT and dopaminergic system function in males and females is important for understanding the full spectrum of MDD etiology. By testing the effects of a range of CORT doses in males and females, we will better understand potential sex differences (or similarities) in CORT’s effects on dopaminergic system function and behavior.

Our findings inspire two related questions regarding CORT’s effects on females: 1) how does chronic CORT treatment decrease DMS dopamine content in females, and 2) why are females resistant to changes in DAT function? CORT treatment’s lack of effect on DMS DAT function in females is likely not due to an interaction with the estrous cycle, as the estrous cycle modulates dopamine reuptake in the ventral, but not dorsal, striatum [53, 64]. Female resistance to CORT-induced impairments in DAT function could be due to their faster metabolism of CORT [65], which could change how chronic CORT treatment impacts gene expression changes in dopamine neurons (among other cell types) through pharmacodynamic differences in glucocorticoid receptor activation. Future studies are needed to address glucocorticoid receptor occupancy and downstream signaling changes that may underlie the effects of CORT on DAT function in males, and dopamine content in females. Sex differences in feedback inhibition of the HPA axis could also lead to a variety of complex effects. For example, chronic CORT treatment could lead to sex differences in expression and secretion of corticotropin releasing hormone (CRH), a neuropeptide that mediates HPA axis activity and has been shown to modulate dopaminergic transmission and decrease operant responding for rewards [66–70]. This possibility is supported by previous observations that depressive-like behaviors in females (e.g., immobility in the forced swim test) are less directly dependent on CORT levels than the same behaviors in males [71]. Future studies are necessary to determine if, and how, levels of CRH or other HPA axis-related signaling molecules are affected by CORT pellet implantation in both sexes.

In sum, our studies suggest that impairment of DMS dopaminergic transmission is a key mechanism underlying stress-induced deficits caused by CORT dysregulation. Our studies lay the groundwork for further dissecting the relationship between CORT signaling and dopaminergic circuit function. It will also be interesting to investigate how chronic CORT treatment affects downstream DMS circuit function and corticostriatal plasticity to sustain the effects of chronic CORT treatment [72]. The more we elucidate these pathways, identifying common mechanisms as well as sex differences, the more we will progress towards new therapeutic approaches for stress-related psychiatric disorders such as MDD.

## Supplementary information

Supplemental Methods and Figures
